# n-Heptane isomerization activities of Pt catalyst supported on micro/mesoporous composites

**DOI:** 10.1186/s13065-021-00787-6

**Published:** 2021-12-03

**Authors:** Z. Ghaderi, M. H. Peyrovi, N. Parsafard

**Affiliations:** 1 Department of Physical and Computational Chemistry, Faculty of Chemistry Science and Petroleum, Shahid Beheshti University, 1983963113 Tehran, Iran; 2 Department of Applied Chemistry, Kosar University of Bojnord, North Khorasan, Iran

**Keywords:** n-heptane isomerization, Catalyst, Catalytic performance, MCM48-HZSM5

## Abstract

Pt loaded on a series of MCM-48 silica and composites with HZSM-5 zeolite, HY zeolite, or TiO_2_ has been prepared and studied for n-heptane isomerization reaction at 200–350 °C. The structural characterization, acid distribution, and morphology of these catalysts were characterized by X-ray diffraction, Fourier transform infrared, UV–Vis diffuse reflectance, scanning electron microscope, temperature**-**programmed desorption of NH_3_, and nitrogen adsorption–desorption methods. The results show that these catalysts have a good selectivity to multi branched isomers, while producing low aromatic compounds. Also, these new composite catalysts prove the catalytic stability during the time of reaction. The most desirable results, and significantly higher n-heptane conversion and isomerization selectivity were achieved with Pt/MCM48-HZSM5 catalyst.

## Introduction

Current stringent environmental protection regulation has specified requirements for reducing the aromatic hydrocarbons proportion that is harmful to the environmental and human health [1–4]. To avoid reducing the gasoline octane number with a decrease in aromatic hydrocarbons, n-heptane isomerization to branched isomers was introduced as a cost-effective reaction in the oil refining industry [5]. The isomerization reaction is thermodynamically and stoichiometrically free of hydrogen, but experiments have shown that in the absence of hydrogen, coke forms rapidly on the catalyst. Therefore, all isomerization units operate under hydrogen pressure [6, 7].

The isomerization catalysts are usually two-factor and include acidic and metallic sites. Metallic sites catalyze dehydrogenation and hydrogenation reactions while acidic sites catalyze isomerization and hydrocracking reactions. The ideal catalyst should have the right balance between acidic and metallic sites and needs to be developed to prevent n-heptane from cracking while promoting the conversion of n-heptane to multi-branched isomers [8].

Three-dimensional structures have received more attention in recent years because these pores in catalysis and separation technologies have significant advantages. The pores of the MCM-48 structure are three-dimensional. These structures have special properties [9]. The symmetry of the siliceous MCM-48 is Ia3d [10].

Although MCM-48 silica has few active sites, the acidity of the surface of siliceous MCM-48 can be effectively improved. Solid micro-mesoporous composite materials have several advantages as catalysts, including strong acidity, good catalyst surface area and other textural properties, selectivity, stability, and environmental friendliness [11].

Though, composite catalysts have been investigated in various catalytic applications such as alkylation, olefin aromatization, benzene oxidation, and cracking [12–14], Pt/MCM48-x (x  =  HZSM-5 zeolite, HY zeolite, or TiO_2_) composite materials have not been synthesized and used in the isomerization of n-alkanes.

In the current work, n-heptane isomerization on a series of Pt/MCM48-x (x  =  HZSM-5 zeolite, HY zeolite, or TiO_2_) catalysts was studied and compared with Pt/MCM-48. The effects of HZSM-5 zeolite, HY zeolite, and TiO_2_ on the catalytic isomerization reaction were examined. The reasons for the improvement of activity, selectivity, stability, coke deposition, and octane number of these catalysts in n-heptane isomerization reaction were discussed at the temperature range of 200–350 °C.

## Experimental

### Preparation method

The mesoporous MCM-48 had synthesized by the following method [15]. Two point four g cetrimonium bromide (CTAB) had added to 50 g of DI water and then dissolved in it. Then, 50 mL ethanol and 12 mL ammonia (32 wt.%) had added to the mixture and stirred for 10 min. Afterward, 3.4 g tetraethyl orthosilicate (TEOS) had slowly added into the above mixture and stirred at room temperature for 2 h. The resulting mixture had filtered, washed with water, and dried in the air. Finally, the obtained MCM-48 silica had calcined for 6 h at 800 °C.

The mesoporous composite catalysts had prepared by the following method [11]. To prepare composites, 0.5 g of HZSM-5 zeolite (or HY zeolite or TiO_2_) had added to the obtained solution during the synthesis and after adding tetraethyl orthosilicate and preparing the homogeneous solution. The final synthesized composites had dried in an oven at 110 °C for 24 h and then calcined at 500 °C for 4 h.

Moreover, Pt (0.6 wt.%) catalysts had prepared by impregnating the support with an appropriate concentration of H_2_PtCl_6_. Then, they had filtered and dried at 110 °C overnight. Eventually, these catalysts had calcined at 300 °C for 4 h.

### Characterization tests

The synthesized catalysts had characterized using various methods. The methods used are briefly mentioned.

X-ray diffraction analysis (XRD) had recorded with an X-PERT diffractometer with Ni filter and graphite monochromator. This device uses Cu kα radiation (wavelength  =  1.15 Å) as an X-ray source. The scan area is 2θ  =  1º–80º at 45 kV and 50 mA with a 0.06° 2θ-step and 1 s per step.

Fourier transform infrared (FTIR) spectroscopy had used for the identification of chemical bonds in the prepared catalysts. FTIR spectra had recorded on a BOMEM FTIR spectrophotometer model Aride-Zone TM, MB series, in the wavenumber of 400–4000 cm^−1^.

UV–Vis diffuse reflectance (UV–Vis DRS) had carried out on a Shimadzu UV-2100 UV–Vis spectrophotometer using BaSO_4_ as a reference. The power catalysts had evaluated in 200–800 nm at room temperature.

To characterize the surface areas and porosity, N_2_ adsorption–desorption isotherms had obtained using the outgassed samples under vacuum at 623 K for 10 h before the measurements. The specific surface area (S_BET_) and the volume of the adsorbed monolayer (V_p_) had evaluated by the BET equation and by assuming an N_2_ molecule to cover 0.162 nm^2^, respectively. The Barret-Joyner-Halenda (BJH) method had also used for calculating the average pore diameter (d_p_).

To investigate the amounts of acidic sites, temperature**-**programmed desorption of NH_3_ (NH_3_-TPD) had carried out with a TPD/TPR analyzer (2900 Micromeritics) instrument.

The morphology of catalysts had investigated on a HITACHI-SU3500 with an accelerating voltage of 30 kV. The amount of coke poisoning had been investigated for all catalysts prepared by coke burning.

### Catalytic evaluation

Catalytic isomerization of n-heptane (n-C_7_) was performed in a continuous fixed**-**bed micro-reactor with 0.2 g of catalyst. The temperature range of this reaction was 200–350 °C and the pressure of hydrogen gas was P  = 1 atmosphere. The catalysts were pretreated at 400 °C for 2 h in an H_2_ flow (40 mL min^−1^). The n-heptane (n-C_7_) was fed into the reactor by a syringe pump with a 1 mL h^−1^ flow rate and mixed with the H_2_ stream at various temperatures. Hydrogen was also introduced in the optimized amount of desired products (H_2_/HC  = 7). The performance of the catalysts was tested after 1 h on stream (TOS) at noted temperatures for each experiment. The catalytic performance of all samples was also investigated at 300 °C for 6 h on stream for studying the coke deposition. Then, the obtained products were taken into account and injected directly and periodically in a gas chromatography with flame ionization detection (an Agilent Technologies 7890A).

In the present work, conversion of n-C_7_ (X_C7_) is calculated by subtracting the remaining amount of n-C_7_ [(C_7_)_f_] determined via GC from unity;

1 $$ {\text{X}}_{{{\text{C}}7}} \left( \% \right) = [1 - \left( {{\text{C}}_{7} } \right)_{f} ] \times 100 $$

The selectivity to each product was also calculated by the following equation;

2 $$ {\text{S}}_{{\text{x}}} \left( \% \right) = \frac{{{\text{n}} - {\text{C}}_{{7{ }}} {\text{transformed into each product}}}}{{1 - \left( {{\text{C}}_{7} } \right)_{{\text{f}}} }} \times 100 $$

## Results and discussion

### Physico-chemical properties

**Fig. 1:**
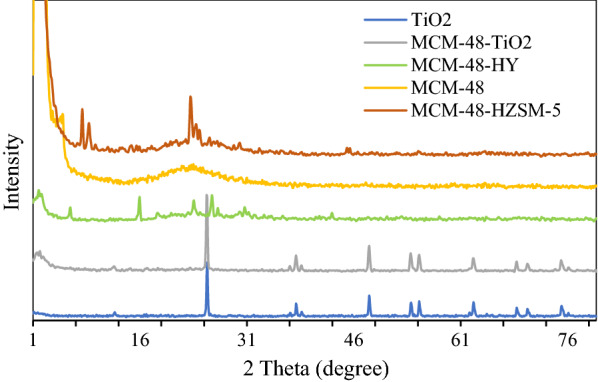
XRD patterns of the Pt synthesized catalysts

The powder XRD patterns of the calcined catalysts are shown in Fig. 1. MCM-48 exhibit**s** the characteristic peaks of the cubic Ia3d symmetry [16, 17]. The main peaks of this mesoporous between 2θ  = 2°–3° can be seen clearly. The sharp peak at 2θ  =  2.9° demonstrates the d211 diffraction of the MCM-48 cubic phase and peak at 2θ  =  3.4° is related to the d220 reflection [18, 19] and at 6.16°, 15.88°, 26.08°, and 30.7° characterize the HY zeolite.

The HZSM-5 zeolite shows sharp peaks in the range of 6–11 degrees and 22–25° [20]. XRD patterns exhibited strong diffraction peaks at 27°, 36°, 48°, and 55° indicating TiO_2_ [21].

**Fig. 2:**
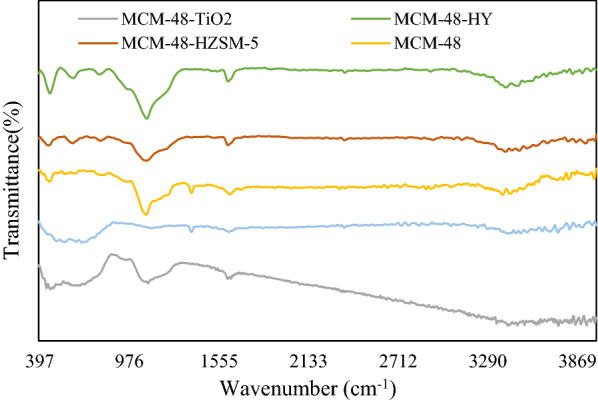
The FT-IR spectra of the Pt synthesized catalysts

Figure 2 shows the infrared spectra for synthesized catalysts. The FT-IR spectra of the MCM-48 and composites present a vibration band near 3400 cm^−1^, which is assigned to ʋ_OH_(H_2_O).

The absorption band near 1639 cm^−1^ is attributed to σ_OH_(H_2_O) [22], and the absorption bands near 1234 cm^−1^ and 1080 cm^−1^ are attributed to ʋ_as_(Si–O–Si) [23]. The zeolite of HZSM-5 has bands at 1024 and 812 cm^−1^. Particularly, the absorption at 554 cm^−1^ is assigned to the five- and six-membered rings of T-O-T (T  =  Si or Al) in the HZSM-5 zeolite [14]. Also, the band observed at 453 cm^−1^ was due to the tetrahedral Si–O bending mode. The FT-IR spectrum of TiO_2_ showed the absorption band at 1631 cm^−1^ to bending vibration of O–H, related to water moisture and 3454 cm^−1^ is related to stretching vibration. The characteristic peak of TiO_2_ was seen at 690 cm^−1^ [24]. About HY zeolite, the bands in the range of 500–950 cm^−1^ due to the zeolite formation structure and the bands near 1630 cm^−1^, refer to the presence of water molecules.

**Fig. 3:**
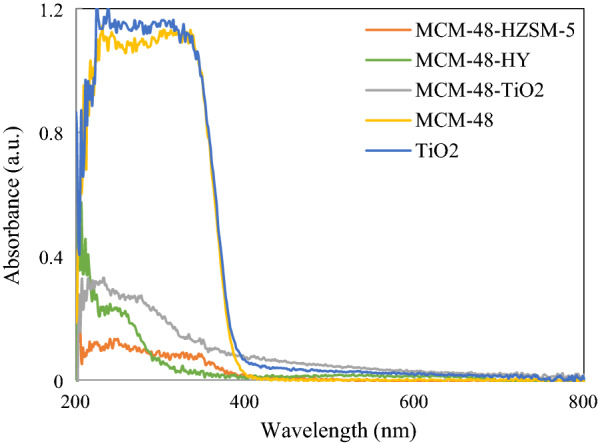
UV–vis DRS of the Pt synthesized catalysts

UV–vis diffuse reflection spectra of synthesized catalysts are shown in Fig. 3. This figure shows three bands for catalysts. The band near 260 nm is difficult to characterize. Pt^4+^ is a low spin d^6^ species that has d-d transfer and ligand to metal charge transfer (LMCT) bands. We tentatively presume that this is a charge transfer (CT) band (oxygen to the metal) [25, 26]. This band can be seen in all spectra. The bands at near 350 and 400 nm must be due to a d–d transition band of Pt [27]. According to the results, Pt metal strongly interacts with the support.

The desorption temperature ranges and quantitative mole number of acid sites (NH_3_-TPD results) are listed in Table 1.

**Table 1:** Physicochemical properties of Pt synthesized catalysts

Catalysts	MCM-48	MCM-48-HZSM-5	HZSM-5	MCM-48-TiO_2_	TiO_2_	MCM-48-HY	HY
Acidity (micro-mol NH_3_/g)							
96.5–325 °C	5.3	223.6	210.0	20.8	21.2	73.7	109.2
325–802 °C	177.5	311.4	360	93.2	30.7	95.3	405.1
L + B	182.8	535.0	570.0	114.0	51.9	169.0	514.3
B/L	33.5	1.4	1.7	4.5	1.4	1.3	3.7
Surface properties							
S_BET_ (m^2^/g)	1237.3	694.7	453.1	213.8	11.1	250.6	689.4
V_p_ (cm^3^/g)	0.750	0.510	0.290	0.405	0.080	0.414	0.160
d_p_ (nm)	2.44	2.94	< 10^–3^ (µ)	7.57	29.63	6.68	1.55

**Fig. 4:**
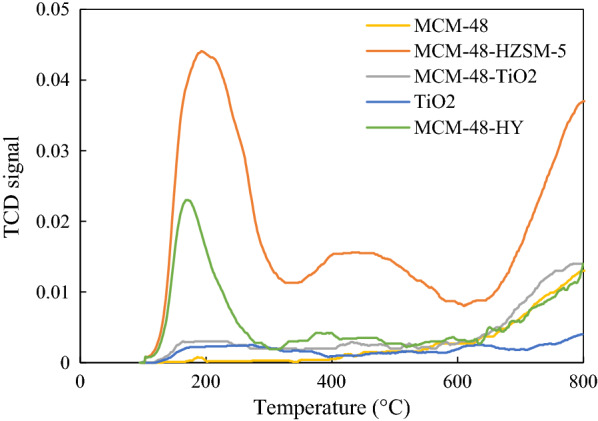
NH_3_-TPD profiles for Pt synthesized catalysts

Figure 4 also shows the acid sites distributions in the siliceous MCM-48 and other samples which are determined by NH_3_-TPD.

The studies [28] have shown that the desorption peak at low temperature corresponds to the weak acid sites, the desorption peak at high temperature corresponds to strong acid sites, and the number of acid sites is related to the peak area. The summarized results in Table 1 show that the number of strong acid sites on the MCM48-HZSM5, MCM48-HY, and MCM48-TiO_2_ are larger than that of the pure MCM-48 and are smaller than that of the HZSM-5 zeolite sample. Also, the number of weak acid sites on all catalysts is larger than MCM-48 silica. Figure 4 presents that the maximum of TPD diagram for MCM-48 shifts to the high temperatures with increasing HZSM-5 zeolite, HY zeolite, or TiO_2_.

**Fig. 5:**
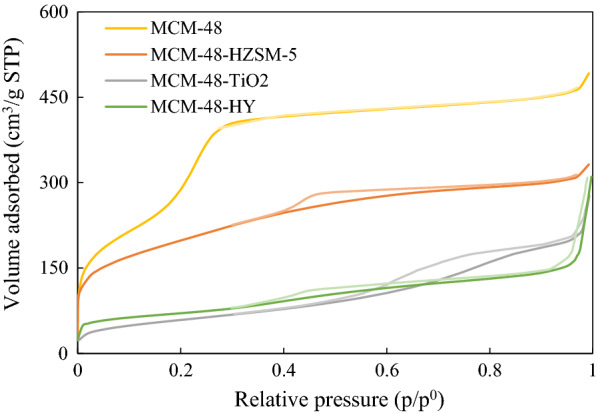
N_2_ adsorption and desorption isotherms for Pt synthesized catalysts

Figure 5 show**s** the N_2_ adsorption–desorption isotherms of the siliceous MCM-48 and other catalysts. The curves of samples conform to the typical Langmuir IV adsorption isotherms (ICPU) accompanied by an H1-type hysteresis loop, which is characteristic of cubic MCM-48 mesoporous materials. For comparison, the textural properties of the composite materials are presented in Table 1. Results show that MCM-48 and composite catalysts have good S_BET_. The pore volume (V_p_) for all synthesized catalysts is smaller than V_p_ for MCM-48. Also, d_p_ in HZSM-5 zeolite and HY zeolite increased after composite formation.

**Fig. 6:**
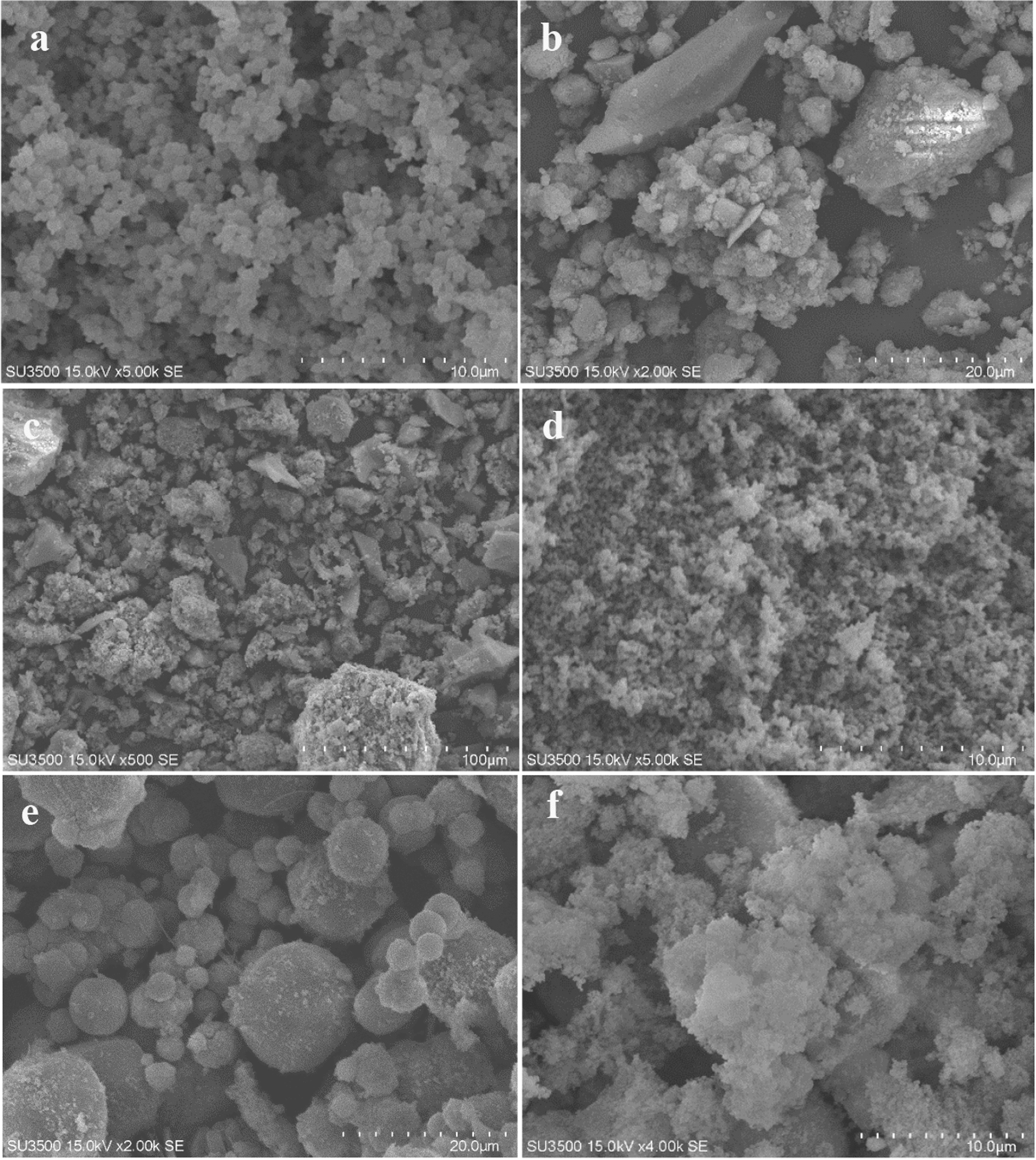
SEM images for various Pt catalysts, **a** MCM-48, **b** MCM48-HZSM5, **c** MCM48-TiO_2_, **d** TiO_2_, **e** HY, and **f** MCM48-HY

Figure 6a–f demonstrates the scanning electron microscope (SEM) images of the Pt/MCM-48, Pt/MCM48-HZSM5, Pt/MCM48-TiO_2_, Pt/TiO_2_, HY, and Pt/MCM48-HY catalysts, respectively. It can be seen from Fig. 6 that the SEM image of siliceous MCM-48 is spherical morphology, and it was in particle size. As can be seen from Fig. 6, the particle size of composite catalysts is not uniform.

### *Catalytic isomerization of n-C*_*7*_

The experimental data have been measured on Pt**-**loaded MCM-48, MCM-48-HZSM-5, HZSM-5, MCM48-TiO_2_, TiO_2_, MCM48-HY, and HY. The catalyst activities were evaluated by the isomerization performance of n-C_7_ over these catalysts between 200 and 350 °C in Table 2. From the conversion results, it is seen that raising the reaction temperature to 350 °C increased almost linearly the n-C_7_ conversion. The highest conversion appeared for the Pt/MCM48-HZSM5 catalyst.

**Table 2:** Catalytic activity, selectivity, coke amount, and RON at various reaction temperatures over different prepared Pt catalysts

Catalyst	T/°C	Coke (%)	Conv. (%)	MOB	MUB	i-C_7_	Crack	Arom	Hydro	RON
MCM-48	200		5.4	8.0	17.7	25.7	0.7	0.3	73.2	7.6
	250		8.7	3.6	10.7	14.3	2.3	0.5	82.9	11.8
	300	4.5	14.6	1.7	5.1	6.8	8.4	0.6	84.2	16.2
	350		19.6	1.1	3.8	5.0	18.4	4.9	71.6	11.0
MCM-48-HZSM-5	200		60.5	11.8	26.0	37.8	12.2	3.3	46.6	50.2
	250		74.6	8.3	20.4	28.7	13.8	4.0	53.5	56.5
	300	6.3	87.7	4.9	12.7	17.2	14.6	5.9	61.7	58.5
	350		95.7	2.3	8.0	10.4	14.8	7.5	67.2	58.6
HZSM-5	200		48.9	11.2	16.0	27.2	0.2	0.1	72.5	8.1
	250		59.8	8.0	7.5	15.5	40	0.3	44.2	20.3
	300	1.8	71.5	5.7	6.0	11.7	42	0.5	45.8	36.8
	350		84.0	3.0	4.6	7.6	43	3.8	45.6	40.9
MCM-48-TiO_2_	200		31.0	14.1	13.1	27.2	16.2	0.3	56.3	23.0
	250		38.4	5.3	11.0	16.3	20.1	0.8	62.8	23.9
	300	9.3	45.4	4.4	4.5	8.8	21.1	1.5	68.5	25.0
	350		59.8	0.3	0.9	1.3	23.0	1.7	74.0	29.4
TiO_2_	200		8.25	6.2	13.1	19.3	0.8	0.9	78.9	11.4
	250		11.8	5.2	8.7	14.0	4.6	1.1	80.4	14.5
	300	18.6	19.4	3.1	5.3	8.5	10.9	1.8	78.8	18.6
	350		24.0	2.2	3.0	5.1	12.8	2.6	79.5	20.6
MCM-48-HY	200		35.6	9.1	20.3	29.5	14.9	0.8	54.8	29.1
	250		47.7	7.6	11.8	19.5	17.5	2.1	60.9	31.8
	300	2.3	54.7	5.9	8.7	14.6	18.1	3.2	64.1	33.4
	350		70.2	1.1	4.3	5.4	19.5	3.7	71.3	39.2
HY	200		28.7	9.1	14.3	23.4	8.0	0.9	67.6	31.1
	250		41.4	5.6	7.9	13.5	16.2	4.6	65.6	26.5
	300	0.1	54.6	3.6	4.9	8.4	17.3	5.5	68.7	31.2
	350		67.8	2.4	3.5	5.9	18.2	5.3	70.6	37.1

It was observed that the catalytic activity follows several factors, including surface properties, structural regularity, metal function, distribution, geometry, strength, type of acid sites, and others. Also, the results of the selectivity to i-C_7_ (mono  +  multi)-, MoB-, MuB branched were shown in Table 2.

These results show that at low conversion or low reaction temperature, the selectivity iso-heptane (i-C_7_) for all the catalysts is high. Because the isomerization reaction has a thermodynamic limit. The ratio of multi-branched to mono-branched iso-heptane (R) nearly range**s**, between 1.0 and 3.0. According to the literature, the formation of MuB isomers is often difficult. Since the molecular size of the multi-branched isomers is larger than of the mono-branched ones, hence, their formation, desorption, and diffusion inside the small pores would be hindered in most of the catalyst, which usually results in more cracking products. But results show that our catalysts have good selectivity to MuB isomers. These results indicate that the pore diameter of our catalysts plays an effective role in the multi-branched C_7_ formation. The ratio of multi-branched to mono-branched iso-heptane (R) at MCM48-HZSM5 and MCM48-HY is larger than that of the HZSM-5 zeolite and HY zeolite Because V_p_ and d_p_ have increased with the formation of the composite. Table 2 demonstrates the selectivity of cracking, aromatization, and hydrogenolysis products of n-C_7_ against the reaction temperatures. As can be seen in Table 2, the Pt/MCM48-TiO_2_ and Pt/TiO_2_ form a little aromatization product. However, this amount is not very low in other catalysts, indicating that aromatization occurs usually via a bifunctional mechanism [29]. Kinds of literatures show that the presence of mesoporosity leads to facilitated hydrogen transfer reactions between olefinic and cyclic intermediates. Therefore, aromatization and cracking are the major reactions of all mesoporous samples. These reactions lead to increase yields of aromatics and LPG. Also, an increase in temperature leads to an increase in cracking and aromatic products. These reactions are competing for reactions and are affected by acidity, type of acid location, geometry, and balance between acid and metal functions. The results show that our synthesized catalysts have a high selectivity for desirable products such as isomerization and aromatization products. As shown in Table 2, the formation of aromatics with these composite catalysts is very low. Aromatic compounds have a high octane number in gasoline. But due to the environmental regulations, at present, the production of reformulated gasoline with low content of benzene is one of the main purpose**s** of the petrochemical industry [30]. To increase octane, the isomerization of n-paraffins is one of the beneficial reactions. As a result, we reported the effects of temperature and catalyst type on the research octane number (RON).

**Fig. 7:**
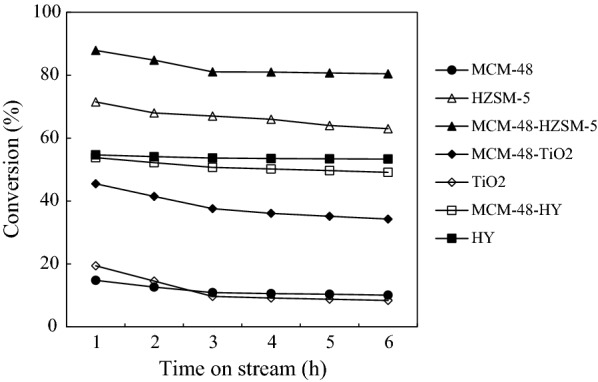
n-C_7_ conversion (%) vs. TOS (h) at 300 °C

Table 2 presents the research octane number (RON) of the reaction for each catalyst. To calculate RON, Eq. (1) was used [26]:

3 $$ {\text{RON}} = \mathop \sum \limits_{{{\text{i}} = 1}}^{{\text{k}}} {\text{y}}_{{\text{i}}} {\text{RON}}_{{\text{i}}} $$

where RON is the measured octane number for total hydrocarbon products, while RONi represents the pure-component octane number for each molecule i in the product, and y_i_ is the volume fractions of reaction products. The results show that Pt/MCM48-HZSM5 at 350 °C compared to other catalysts, due to the production of molecules with higher RON_i_, provides higher RON. The resistance against hydrocarbon poisoning for one catalyst as well as possible changes in the catalyst activity and selectivity of various products has been explored. To assay the stability of synthesized catalysts in the range of reaction time, the experiments were continued for 6 h. A graph of conversion vs. time on stream (TOS) at 300 °C for all catalysts is given in Fig. 7. According to the figure, changes in the reaction characteristics usually happened in the first 1–2 h on stream. According to the results, the conversion of these catalysts decreases with increasing TOS. But the decrease in the case of Pt/TiO_2_ is higher than other composite catalysts. The Coke formation on the catalysts was investigated to further comprehend the results. The amount of coke poisoning was evaluated for all catalyst**s** prepared by coke burning. The catalysts, which were tested for 6 h at 300 °C, were placed in an oven at 120 °C to lose moisture, then weighed and placed in an oven at 300 °C for one hour. Immediately after cooling, the catalyst was weighed again to obtain the weight difference. The weight difference is the amount of coke that is given in Table 2.

This table shows that the coke formation on the Pt/HY catalyst is lower than the other catalysts, while Pt/TiO_2_ has a higher coke content. This more amount of coke on Pt/TiO_2_ catalyst can be confirmed with the lower stability after 6 h of reaction and previous results for surface and acidity properties. The balance between the acidity of the catalyst and the surface properties and metal sites has caused that this catalyst has more coke.

## Conclusions

The results show that the mostly synthesized catalysts have a suitable conversion, selectivity, and stability for isomerization of n-C_7_. The micro/mesoporous composite catalysts have a micropore and mesopore dual pore size distribution. The HZSM-5 zeolite, HY zeolite, and TiO_2_ influence the structural regularity and surface acidity of MCM-48. The Pt/MCM48-HZSM5 catalyst shows suitable catalytic activity in the isomerization of n-C_7_. The coke deposition induces a decrease of the catalytic activity, especially in the first 1–2 h. The synthesized catalysts have suitable selectivity to MuB isomers and aromatic products. The porosity, metal dispersion, acidity, and nature of the acid site have led to the good performance of these catalysts. The results show that the synthesized catalysts have led to high octane number of gasolines, however, this result will improve with future studies.

## Data Availability

All data generated or analyzed during this study are included in this published article.
